# Extremophilic Prokaryotic Endoxylanases: Diversity, Applicability, and Molecular Insights

**DOI:** 10.3389/fmicb.2021.728475

**Published:** 2021-09-09

**Authors:** Digvijay Verma

**Affiliations:** Department of Environmental Microbiology, Babasaheb Bhimrao Ambedkar University, Lucknow, India

**Keywords:** prokaryotes, extremozymes, endoxylanases, lignocelluose, biobleaching

## Abstract

Extremophilic endoxylanases grabbed attention in recent years due to their applicability under harsh conditions of several industrial processes. Thermophilic, alkaliphilic, and acidophilic endoxylanases found their employability in bio-bleaching of paper pulp, bioconversion of lignocellulosic biomass into xylooligosaccharides, bioethanol production, and improving the nutritious value of bread and other bakery products. Xylanases obtained from extremophilic bacteria and archaea are considered better than fungal sources for several reasons. For example, enzymatic activity under broad pH and temperature range, low molecular weight, cellulase-free activity, and longer stability under extreme conditions of prokaryotic derived xylanases make them a good choice. In addition, a short life span, easy cultivation/harvesting methods, higher yield, and rapid DNA manipulations of bacterial and archaeal cells further reduces the overall cost of the product. This review focuses on the diversity of prokaryotic endoxylanases, their characteristics, and their functional attributes. Besides, the molecular mechanisms of their extreme behavior have also been presented here.

## Introduction

**FIGURE 1:**
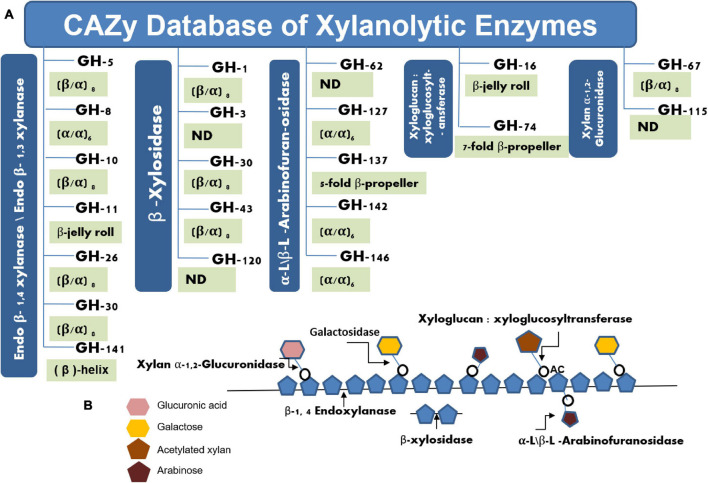
Pictorial representation of various glycosyl hydrolases families having xylan hydrolytic enzymes **(A)**. Schematic representation of various xylanolytic enzymes for their activity toward heterogeneous xylan **(B)**.

The natural biomass of lignocellulose finds potential as an alternate energy source that exhibits the possibility to replace the nonrenewal energy sources. Lignocellulose is chiefly composed of cellulose (40%), hemicellulose (33%), and lignin (23%) (Ninawe et al., 2008). Therefore, this natural biomass shares a significant proportion of sugars that can be used for bioethanol conversion as well as for several other biotechnological applications (Collins et al., 2005). However, the creeping growth in this technology is not commendable as would have been expected. Of the several reasons, the dearth of industrially fit enzymes/biocatalysts is one of the major reasons to lose this goal. The lignocellulolytic enzymes significantly participate in the conversion of complex biomass into valuable products (Bocchini et al., 2002; Cordeiro et al., 2002). Industries are primarily interested in cellulosic fibers for making quality papers (Collins et al., 2005; Bhardwaj et al., 2019) or for extracting sugars for bioethanol production (Bhalla et al., 2015; Xiao et al., 2019), where endoxylanases facilitate this process by removing xylan polysaccharides. Besides, the outstanding applications of xylooligosaccharides (XOs) as prebiotic further grab the attention to harvest this valuable natural resource (Dornez et al., 2011). At present, the CAZy database^1^ comprises 4,321 bacterial and 44 archaeal glycosyl hydrolase family 10 (GH-10) type xylanolytic enzymes, whereas the GH-11 family harbor 1,362 bacterial and only 11 archaeal representatives. Both of these xylanases share several common features, where both exhibit glutamate as catalytically important residues. However, amino acid compositions, molecular weight, 3D structures, and signature sequences categorize them in different families (Figure 1A). The GH-11 family xylanases are considered true xylanases due to their substrate specificity toward xylan (Paes et al., 2012; Yan et al., 2021).

Xylan is the main constituent of the hemicellulosic compounds that accounts for one-third of total organic carbon present on earth (Sharma et al., 2007; Kumar et al., 2013b). Therefore, not only does xylan act as a reservoir of sugars but also their degradation facilitates the release of cellulosic fibers from lignocellulosic material (Collins et al., 2005). In industries, this process occurs at very high temperatures and pressure under alkaline conditions to remove the xylan component from lignocellulosic material (Kumar and Satyanarayana, 2012). Such processes are energy-intensive and add a significant chunk of pollutants in the environments such as chlorine, dioxins, and other air pollutants (Sharma et al., 2017). Therefore, hemicellulases are preferred to reduce the harmful effects that occur during the chemical and mechanical treatments of hemicellulosic materials to make the process eco-friendly and cost-effective (Collins et al., 2005). Due to the extreme conditions of the industries, hemicellulases of extreme properties have always been in demand in industries for their applications. Hemicellulases are the bundle of enzymes that include endoxylanases, β-xylosidase, arabinofuranosidase, and acetyl-xylan esterase, among others (Bhardwaj et al., 2019). Endo (EC: 3.2.1.8) represents the chief enzyme of hemicellulases that cleave the β-glycosidic bonds of the xylan backbone and release XOs as a product, while the β-xylosidases (EC: 3.2.1.37) act on xylobiose or other xylooligosaccharides to generate monomeric sugar xylose as the final product. Arabinofuranosidases (EC: 3.2.1.55) and acetyl xylan esterases (EC: 3.1.1.72) attack on side chains of heterogeneous xylan substrate and assist xylanases and β-xylosidases for the complete degradation of xylan (Collins et al., 2005; Figure 1B). The synergistic effect of these enzymes removes the xylan as well as lignin from the cellulosic fibers in a specific and refined manner without affecting the quality of the cellulose (Sharma et al., 2007). In search of efficient enzymes, extremophilic microorganisms always come on the first choice as extremophiles are enough capable to perform this task due to the abundance of proteins or enzymes of extreme properties (Verma and Satyanarayana, 2020). These astonishing microorganisms represent themselves as the pioneer organisms that were evolved during the origin of the earth (Vieille and Zeikus, 2001). Therefore, the study of extremophiles always provokes researchers to reveal their hidden properties and the inherent mechanisms for their survival in extreme environments (Basit et al., 2018; Merino et al., 2019). The proteins and enzymes have been reported to play a pivotal role in acquiring adaptations to several extremophiles. Such extremozymes exhibit several unique features that have been accumulated during evolution (Vieille and Zeikus, 2001). Several such enzymes were extensively explored in numerous ways to understand their mechanisms as well as for their applicability in several industries. Hemicellulosic extremozymes, therefore, enhance the interest due to acquired properties of their natural habitat. Although the hemicellulose-degrading enzymes have been discovered from bacteria to fungi, the majority of these enzymes have been of mesophilic origin (Collins et al., 2005; Bhardwaj et al., 2019). Such enzymes are not able to withstand the extreme conditions of the industries and deactivate during their employment in harsh conditions (Yang et al., 2017; Xiang et al., 2019). For example, xylanases find applicability in the bleaching of pulp samples in the paper industry (Viikari et al., 1986). However, the bleaching process of pulp in the paper industry occurs at very high temperature (>90–105°C) and pressure (8–10 atmospheric pressure) under alkaline conditions (pH > 8.5–11); therefore, thermo-alkali-stable xylanases are required to meet these extreme conditions (Viikari et al., 1986; Kumar and Satyanarayana, 2012; Ayubi et al., 2021). An enzyme of mesophilic properties cannot perform well in such conditions and gets destroyed easily during the process. Several such requisites of the industries encourage the discovery of extremophilic hemicellulases. Therefore, recovering extremophiles from a specific environment is the first step of finding an enzyme of interest. Traditional cultivation approaches lose a significant portion of 99% of microorganisms of environmental samples (Amann et al., 1995; Handelsman, 2004). This count further enhances in the case of extremophilic microorganisms that are always present in lower count over the mesophilic microbes in any environment. Therefore, the cultivation of the extremophiles in the pure form of isolates is a tedious job that needs huge expertise and patience. Metagenomic approaches offer an alternate way to extract the entire community DNA (bacteria, archaea, and eukarya) of an environmental sample bypassing the cultivation of the microorganisms (Handelsman, 2004). The technique is considered the second most significant invention after the discovery of the microscope. The technique has been successfully employed for retrieving several novel genes of microbial enzymes and other bioactive molecules from diverse environments including extreme habitats (Ferrer et al., 2005; Prayogo et al., 2020). This review discusses the diversity and applicability of extremophilic endoxylanases in detail and provides molecular insights into the extremophilic xylanases.

## Fondness of Bacterial Endoxylanases Over the Fungal Origin

Hemicellulose-degrading microorganisms show their presence in all three domains of organisms. Prokarya and archaea are the major groups where extremophilic microorganisms are more abundant over eukarya. There are several reasons for considering bacterial endoxylanases over other microbial sources (Zhu et al., 2011; Chakdar et al., 2016; Verma et al., 2019). Easy cultivation and harvesting of bacterial cells along with a short lifespan consider the bacteria an attractive factory for enzyme production (Verma et al., 2019). Besides, the biophysical and biochemical properties of bacterial hemicellulases such as wide pH (3.0–10.0) and temperature (20–105°C) range further broaden their scope for their extensive applications in industries. The majority of the fungal xylanases are either acidic or neutral in their characteristics, while several bacterial xylanases have been reported for their optimum pH under alkaline conditions (Mamo et al., 2006; Amel et al., 2016; Yadav et al., 2018), which is a highly required property for the paper industry in bioleaching of pulp samples. The fungus also produces copolymers along with extracellular enzymes that significantly reduce the overall viscosity of the reaction mixture (Sharma et al., 2016). Moreover, bacterial hemicellulases are comparatively of low molecular weight over the fungal origin. Low molecular weight (<30 kDa) hemicellulases diffuse rapidly into the rigid lignocellulosic biomass and thus enhance their oxygen penetration rate during the downstream processing over the high molecular weight (>30 kDa) homologs (Breccia et al., 1998; Sharma et al., 2016; Verma et al., 2019). Several fungal xylanases come along with cellulase activity (Subramaniyan and Prema, 2002; Polizeli et al., 2005) that sometimes is not apt for many industries, while the majority of the bacterial xylanases are devoid of such activity and therefore more xylan-specific over the fungal endoxylanases (Robledo et al., 2016). Fungal endoxylanases are being employed in the industries; it may be due to their high production over the bacterial origin, else bacterial endoxylanases provide better alternates than fungi. Fungal endoxylanases may be preferred when the reaction conditions are not extreme and only highly active endoxylanases are required. Of various extreme properties, thermostability is the chief concern of several industries. Many thermophilic (45–65°C) and hyperthermophilic (>65°C) bacteria have been reported to exhibit thermophilic endoxylanases. For instance, *Thermotoga* (Bok et al., 1994; Winterhalter et al., 1995; Shi et al., 2013), *Clostridium* (Rani and Nand, 2000; Heinze et al., 2017), *Caldicellulosiruptor* (Luthi et al., 1990), *Geobacillus* (Sharma et al., 2007; Kumar et al., 2013b; Verma et al., 2013a), *Bacillus* (Mamo et al., 2006; Kumar et al., 2013a), and *Streptomycetes* (Bajaj and Singh, 2010) are the major genera for exhibiting thermophilic endoxylanases. The majority of these bacteria have been isolated from thermophilic habitats, which could be the reason for showing the reflection of their environment in their properties (Saleem et al., 2012; Verma et al., 2019; Irfan et al., 2020). However, there are reports on endoxylanases from those bacteria that have not been isolated from extreme environments, even then exhibiting extreme properties. Members of *Geobacillus* are the wonderful examples of such bacteria that uncovered several enzymes of odd characteristics that significantly vary from their environmental conditions (Zeigler, 2014). A thermophilic endoxylanase of *B. pumilus* B20 was isolated from paper mill soil samples that showed the optimum temperature at 60°C under a slightly acidic pH of 6.5, despite their mesophilic origin (Geetha and Gunasekaran, 2017). Endoxylanase of *Bacillus halodurans* TSEV1 also showed extremophilic properties (Topt. 70°C and pH 9.0) that were discovered from a paper effluent sample (Kumar and Satyanarayana, 2014). Similarly, many endoxylanases of *Pseudomonas* sp. XPB-6 (Sharma and Chand, 2012), *Geobacillus thermoleovorans* (Verma and Satyanarayana, 2012), *Paenibacillus* sp. HPL-001 (Hwang et al., 2010), and *Streptomyces* sp. S27 (Li et al., 2009) were isolated from mesophilic environmental samples that unveiled thermophilic properties on characterization. On the other hand, archaeal endoxylanases have been usually recovered from Crenarchaeotes such as *Thermosphaera aggregans* (Huber et al., 1998), *Acidilobus saccharovorans* (Menasria et al., 2018), and *Sulfolobus solfataricus* (Cannio et al., 2004). On characterization, the majority of these archaeal endoxylanases showed outstanding thermostability at higher temperatures but under acidic conditions.

## Biophysical Properties of Extremophilic Endoxylanases

**FIGURE 2:**
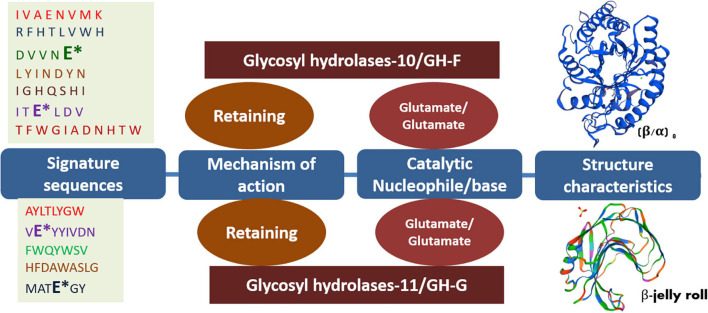
Different features of GH-10 and GH-11 endoxylanases. Asterisk (*) represents the catalytically important glutamate residues in the putative signature sequences of respective GH-10 and GH-11 endoxylanases. The frequency of the amino acids in the signature sequences may slightly vary among the respective families while retaining 100% conservancy for catalytically important residues glutamate.

The GH-10 and GH-11 xylanases of either bacteria, archaea, or fungal origin share several common features such as both exhibiting glutamate as catalytic acid/base in their respective signature sequences and both following the retaining mechanism of catalysis (Figure 2). In both families, several extremophilic endoxylanases have been characterized. Extremophilic properties of hemicellulases make them different from the mesophilic homologs. These enzymes have immense significance in several industries. The thermo-alkali-stable xylanases find potential application in the bleaching of pulp in the paper industry. Similarly, thermo-acid-stable xylanases and xylosidases have employability in the bread, feed, and juice industries. Characterization of such hemicellulases uncovers several biochemical and biophysical properties of hemicellulases (Table 1).

**TABLE 1:** Characteristics of extremophilic endoxylanases from various prokaryotic microorganisms.

Bacteria	Source	GH family	MW (kDa)	Topt.	pHopt.	Km (mg ml^–1^)	Vmax (μM min^–1^ mg^–1^)	References
*Staphylococcus* sp. SG-13	Alkaline soil	-NM-	60	50.0	7.5-9.2	Birchwood: 4 Oat spelt: 7	Birchwood: 90 Oat spelt: 55	Gupta et al., 2000
*Bacillus halodurans* S7	Soda lake	GH-10	43	75.0	9.0-9.5	Beechwood: 5.42 8Birchwood: 4.53 Oat spelt: 4.37	Beechwood: 252 Birchwood: 230 Oat spelt: 319	Mamo et al., 2006
*Glaceocola mesophila* KMM 241	Marine Habitat	GH-10	43.0	30.0	7.0	Beechwood: 1.22 Birchwood: 1.42 Oat spelt: 3.80	Beechwood: 98.31 Birchwood: 86.70 Oat spelt: 60.00	Guo et al., 2009
*Streptomyces* sp. S27	Soil	GH-11	27–45	60.0	7.0	Oat spelt xylan: 6.2 Birchwood xylan: 2.3	Oat spelt xylan: 9930.5 Birchwood xylan: 4962	Li et al., 2009
*S. thermocyaneoviolaceus*	Korean Culture Center of Microorganisms	-NM-	35	60.0	5.0	Birchwood: 10.88	Birchwood: 3.02	Shin et al., 2009
*Paenibacillus campinasensis*	PCR	GH-11	41.0	60.0	7.0	Oat spelt xylan: 6.78	Oat spelt xylan: 4953	Ko et al., 2010
*Bacillus* sp.	Microbiological Culture Collection Center	GH-10	45	70.0	7.0	Birchwood: 2.53	Birchwood: 600	Zhang et al., 2010
*Paenibacillus sp.* HPL-001	Organic-rich soil	-NM-	38.1	55.0	5.5	Birchwood xylan: 5.35	Birchwood xylan: 199.17	Hwang et al., 2010
			38.1	45.0	9.5	Birchwood xylan: 3.23	Birchwood xylan: 6.95	
*Actinomadura sp.* S14	Compost of Thailand	GH-11 XynS14 (*E. coli*)	30-40 (with CBM)	80.0	6.0	Beechwood: 7 Birchwood: 11.2 Oatspelt: 21.6	Beechwood: 1,940 Birchwood: 2,130 Oatspelt: 2,210	Sriyapai et al., 2011
		GH-11 XynS14 (*P. pastoris*)	30-40 (with CBM)	80.0	6.0	Beechwood: 8.9 Birchwood: 12 Oat spelt: 14	Beechwood: 7,520 Birchwood: 8,310 Oatspelt: 7,120	
*Saccharopolyspora pathumthaniensis* S582	Termite gut	GH-10	36	70.0	6.5	Beechwood: 3.92	Beechwood: 256	Sinma et al., 2011
*Bacillus pumilus* ARA	Culture pollutes	GH-11	23	50.0	6.6	Oat spelt: 5.53	Oat spelt (Kcat/Km): 10.14 ml/mg/min	Qu and Shao, 2011
*Bacillus* sp.	Hot spring	-NM-	61	60.0	8.0	Birchwood: 6.5 Oat spelt: 9.5	Birchwood: 53.6 Oat spelt: 40.3	Saleem et al., 2012
*Geobacillus thermoleovorans*	Pulp sample	GH-10	45	80.0	8.5	Birchwood xylan: 2.1	Birchwood xylan: 42.5	Verma and Satyanarayana, 2012
*Pseudomonas* sp. XPB-6	Soil	NM	123	60.0	6.5	Birchwood xylan: 0.60	Birchwood xylan: 144.92 IU/mg	Sharma and Chand, 2012
*Bacillus sp.*	Soil	GH-11	29.8	50.0	8.0	Birchwood xylan: 5.26	Birchwood xylan: 277.7	Kamble and Jadhav, 2012
*Geobacillus thermoleovorans* TSAA1	High-temperature compost	GH-10	50	70.0	9.0	Birchwood: 0.625	Birchwood: 555.5	Verma et al., 2013a
*Bacillus subtilis* BS05	Fermentation lab.	NM	23	50.0	5.0	Birchwood xylan: 1.15	Birchwood xylan: 850 S^–1^	Irfan et al., 2013
*Thermotoga thermarum*	Xylanase gene synthesis	GH-10	130	95.0	7.0	Beechwood: 2.57	Beechwood: 325.32	Shi et al., 2013
*Bacillus halodurans* TSEV1	Paper effluent	GH-10	40	70.0	9.0	Birchwood: 2.0	Birchwood: 488	Kumar and Satyanarayana, 2014
*Paenibacillus* sp. NF		GH-10	37	60.0	6.0	Oat spelt: 5.64 Birchwood: 6.32	Oat spelt: 3364 Birchwood: 3216	Zheng et al., 2014
*Thermotoga thermarum* DSM 5069	–	GH-10	40.5	80.0	6.0	Beechwood: 1.8	Beechwood (Kcat/Km): 289 ml/mg/sec	Shi et al., 2014
*Dictyoglomus thermophilum*		GH-10	20	90.0		Birchwood: 2.8	Birchwood: 1782	Li et al., 2015
*Caldicellulosiruptor bescii*	PCR	GH-10	40.0	70.0	7.2	Beechwood: 1.90 Oat spelt: 1.94 Birchwood: 2.16	Beechwood: 199.2 Oat spelt: 183.4 Birchwood: 148.8	An et al., 2015
*Streptomyces coelicolor* A3(2)	Soil	GH-10	47.0	60.0	6.0	Beechwood: 0.24	Beechwood: 6.86	Enkhbaatar et al., 2016
*Caldicoprobacter algeriensis* THC1(T)	Hydrothermal hot spring	GH-11	35.0	70.0	11.0	Oat spelt xylan: 1.33	Oat spelt xylan: 595	Amel et al., 2016
*Saccharophagus degradans* 2-40	ATCC	GH-10	42.4	30.0	7.0	Birchwood xylan: 10.4	Birchwood xylan: 253	Ko et al., 2016
*Bacillus pumilus* B20	Paper mill soil	NM	85	60.0	2.3	Oat spelt: 2.3	Oat spelt: 50	Geetha and Gunasekaran, 2017
*Anoxybacillus kamchatkensis* NASTPD13	Geyser valley	NM	37	65.0	9.0	Beechwood: 0.7	Beechwood: 66.64	Yadav et al., 2018
*Bacillus licheniformis* DM5	Water	GH-30	38	50.0	6.5	Beechwood: 1.5	Beechwood: 2.7 U/ml	Ghosh et al., 2019
*Bacillus licheniformis S3*	Hot spring	-NM-	-NM-	55.0	6.0	Xylan: 8.6	Xylan: 43.71	Irfan et al., 2020
*Bacillus subtilis strain CAM 21*	-NM-	GH-11	24	50.0	7.0	Birchwood xylan: 2.9	Birchwood xylan: 15207	Monica and Kapoor, 2021

NM, not mentioned.

### Thermophilic Endoxylanases

Thermotogales is one of the orders of bacteria that harbor promising candidates that can withstand extreme environmental conditions. Several endoxylanases of genus Thermotoga have been identified for thermophilic hemicellulases for exhibiting their optimum temperature (Topt.) beyond 70°C. A thermophilic endoxylanase (XynA) of GH-10 family from *Thermotoga* sp. strain FjSS3-B.1 is one of the most thermostable xylanases reported to date that showed maximum activity at 105°C. Several such xylanases from *Thermotoga* spp. such as *T. thermarum* (Shi et al., 2014) and *T*. *naphthophila* (Hamid and Aftab, 2019), *Thermotoga neapolitana* (Zverlov et al., 1996), and *Thermotoga petrophila* (Ikram et al., 2012) have been reported for having hyper-thermophilic xylanases.

*Bacillus* and *Geobacillus* represent another major group of bacteria that exhibit plenty of thermophilic endoxylanases. *Bacillus* such as *Bacillus stearothermophilus* (Topt. 60°C; Nammori et al., 1990), *Bacillus stearothermophilus* SDX (Topt. 70°C; Dhiman et al., 2008), *Bacillus licheniformis* A99 (Topt. 60°C; Archana and Satyanarayana, 1998), *Bacillus pumilus* MK001 (Topt. 60°C; Kapoor et al., 2008), *Bacillus halodurans* (Topt. 60°C; Mamo et al., 2006), *Bacillus amyloliquefaciens* (Topt. 80°C; Breccia et al., 1998), and *Bacillus circulans* D1 (Topt. 60°C; Bocchini et al., 2008) have been reported for thermophilic xylanases. An alkaliphilic species of *B. halodurans* S7 isolated from an Ethiopian soda lake revealed an endoxylanase of polyextremophilic properties by retaining 100% of total endoxylanase activity even after 12 h of incubation at 50°C (Mamo et al., 2006). Another strain of *B. halodurans* isolated from Sambhar Lake, India, also shares similar biophysical properties with an optimum temperature at 70°C (Kumar and Satyanarayana, 2012). Moreover, this recombinant endoxylanase was active in the broad temperature range of 30°C to 100°C, where it showed a half-life of 30 min at 80°C. An unidentified species of *Bacillus* NG-2 has been identified for thermostable endoxylanase that exhibited T_1/2_ of 75 min at the optimum temperature of 70°C (Gupta et al., 2000). Similarly, the endoxylanases of *Bacillus* sp. GRE7 (Kiddinamoorthy et al., 2008) and *B*. *firmus* (Chang et al., 2004) also showed their maximum enzymatic activity at 70°C. A novel GH-10 thermostable endoxylanase of *Bacillus* sp. KW1 (Topt. 65°C) showed activity over a wide range of substrates including carboxymethyl cellulose (CMC) and cellobiose (Wang et al., 2019). Besides, a plethora of enzymes have been documented from several *Bacillus* spp. that find their optimum temperature at or below 50°C such as *B. amyloliquefaciens* (Breccia et al., 1998), *Bacillus stearothermophilus* SSP34 (Subramaniyan and Prema, 2002), *B. amyloliquefaciens* strain SK-3 (Kumar et al., 2017), and *Paenibacillus campinasensis* BL11 (Ko et al., 2010).

*Geobacillus*, another very close genus of *Bacillus* that is usually present in hot and arid soil, has been identified for having endoxylanases of thermophilic characteristics. The member of this genera grabbed huge attention due to the presence of several thermostable proteins and enzymes that find potential biotechnological applications. *Geobacillus thermoleovorans* (Verma and Satyanarayana, 2012), *G. thermoleovorans* AP07 (Sharma et al., 2007), and *Geobacillus thermodenitrificans* TSAA1 (Verma et al., 2013a) are well known for their polyextremophilic endoxylanases. The source of these isolates is majorly from thermophilic environments; therefore, the selection of samples enhances the possibility to get the desired enzymes. A recombinant endoxylanase *G. thermoleovorans* isolated from pulp samples of a paper industry showed its activity over a broad range of temperature from 40 to 100°C with an optimum temperature of 80°C. More interestingly, this enzyme exhibited a half-life of 1 h at 80°C, which makes it suitable for several industries (Verma and Satyanarayana, 2012). A thermophilic bacterium *G. thermodenitrificans* TSAA1 isolated from a high-temperature compost plant in Fukuoka, Japan, was uncovered for a thermostable endoxylanase with an optimum temperature at 70°C along with a half-life of 10 min at 80°C and was successfully employed for saccharifying agroresidues (Verma et al., 2013a). This recombinant enzyme showed high homology with the GH-10 endoxylanases of other close species of *Geobacillus* such as *G. thermodenitrificans*, *G. thermoleovorans*, and *G. stearothermophilus*. Bhalla et al. (2014, 2015) reported the most promising thermostable endoxylanase from *Geobacillus* WSUCF1 that showed outstanding stability of 12 days by retaining 50% of total enzymatic activity at 70°C. The group further claimed that this enzyme is the most thermostable enzyme from any other species of *Geobacillus* to date. Besides, there are several species/strains of *Geobacillus* that find their optimum temperature at or below 60°C such as xylanases of *G*. *thermodenitrificans* (Irfan et al., 2018), *G*. *stearothermophilus* KIBGE-IB29 (Bibi et al., 2018; Mathew et al., 2018), and *Geobacillus* strain DUSELR 13 (Bibra et al., 2018). Although *Geobacillus* are obligate thermophiles (Topt. of growth 55–65°C), even their presence is not limited to thermophilic environments; rather, numerous *Geobacillus* spp. have been isolated from mesophilic as well as low-temperature environments (Zeigler, 2014). Overall, it has been observed that the majority of xylanolytic enzymes recovered from *Geobacillus* spp. exhibit thermostability under acidic as well as alkaline conditions for the longer duration that prove their suitability for various biotechnological applications.

*Clostridium absonum* CFR-702 (Rani and Nand, 2000), *Dictyoglomus* sp. B1 (Adamsen et al., 1995), *Rhodothermus marinus* ITI376 (Dahlberg et al., 1993), and *Thermoactinomyces thalophilus* sub gr. C (Kohilu et al., 2001) are the other bacterial species reported for thermophilic endoxylanases, where xylanase of *Arthrobacter* sp. MTCC 5214 has been reported for exhibiting extreme endoxylanase activity even at 100°C (Khandeparkar and Bhosle, 2006). The genus *Caldicellulosiruptor* also represents hyperthermophilic, Gram-positive anaerobes and has been reported as a reservoir of GH-10 thermostable xylanases (Luthi et al., 1990; Liu et al., 2017; Jia and Han, 2019), *C*. *lactoaceticus* (Topt. 80°C; Jia et al., 2014), and *C*. *owensensis* (T opt. 90°C; Liu et al., 2016). Moreover, these xylanases were stable at higher temperatures for long durations and therefore find suitability in bleaching of pulp samples. For instance, the recombinant xylanase (CoXynA) retains 50% of its total activity even after 1 h of incubation at 80°C (Liu et al., 2018). These enzymes have been successfully employed for the generation of XOs from lignocellulosic biomass. The *Caldicellulosiruptor* is considered as one of the most promising sources of hemicellulases that exhibit an exceptional potential to deconstruct the lignocellulosic material. To further improve the overall lignocellulose-degrading activity of *C. bescii*, two xylanase-encoding genes of *Acidothermus cellulolyticus* were cloned and expressed in *C. bescii* which resulted in a significant increase in the exoproteome of *C. bescii* on xylan substrates (Kim et al., 2016).

A unique GH-10 endoxylanase (XynDZ5) was recovered from a *Thermoanaerobacterium* species isolated from an Icelandic hot spring. On characterization, it was identified as a highly thermostable endoxylanase with optimum activity between 65 and 75°C. Interestingly, it showed very low identity of 26% with the other close homologs (Zarafeta et al., 2020). Similarly, a hyperthermophilic bacterium *Stenotrophomonas maltophilia* produced high molecular weight xylanase that showed fair stability in a wide range of temperature (30–80°C). Thermostable xylanases of *Clostridium absonum* CFR-702 (Rani and Nand, 2000), *Dictyoglomus* sp. B1 (Adamsen et al., 1995), *Clostridium cellulovorans* (Sleat et al., 1984), *Thermopolyspora flexuosa* (Anbarasan et al., 2017), and *Rhodothermus marinus* (Dahlberg et al., 1993) are also notable.

### Alkaliphilic Endoxylanases

The alkalophilicity of endoxylanases is another important parameter of several many industries especially the paper and pulp industry. The majority of the *Geobacillus* spp. produce alkaliphilic hemicellulases. The recombinant xylanases of *G. thermoleovorans* AP07 (pHopt. 8.0; Verma and Satyanarayana, 2012), *G. thermodenitrificans* A333 (pHopt. 7.5; Marcolongo et al., 2015), *G. thermoleovorans* (pHopt. 8.5; Sharma et al., 2007), and *Geobacillus* sp. WBI (pHopt. 6–9; Mitra et al., 2015) have also been well documented for alkaliphilic endoxylanases. However, xylanases of *G. thermodenitrificans* TSAA1 (pHopt. 7.0; Verma et al., 2013a), *G*. strain DUSELR13 (pHopt. 7.0; Bibra et al., 2018), and *G*. s*tearothermophilus* KIBGE-IB29 (pHopt. 6.0; Bibi et al., 2018) behaved differently by showing their optimum pH from slightly acidic to neutral range. Besides optimum pH in the alkaline range, the stability of these xylanases at higher pH makes them eligible to withstand the industrial extreme conditions. The recombinant xylanases of *Geobacillus* sp. WBI (Mitra et al., 2015) and *G*. *thermodenitrificans* A333 (Marcolongo et al., 2015) were highly stable that can withstand the higher pH of 11.0 by retaining 97 and 75% of activity for 1 h, respectively. The endoxylanases from *G. thermoleovorans* (Verma and Satyanarayana, 2012) and *G. thermodenitrificans* TSAA1 (Verma et al., 2013a) were successfully employed for the saccharification of the agro-residues for the liberation of xylo-oligosaccharides. Numerous highly alkaliphilic endoxylanases have been reported from several strains of *B. halodurans* (Mamo et al., 2006; Kumar and Satyanarayana, 2011, 2012) that find their optimum pH 8.0 and above along with fair stability for long hours under high alkaline conditions. A polyextremophilic endoxylanase of *B. halodurans* TSEV1 was successfully employed for the bleaching of kraft pulp at the higher pH of 10.0 (Kumar and Satyanarayana, 2012). This first report of bleaching by endoxylanase of *B. halodurans* significantly reduced the kappa number by 14.6% and enhanced the brightness by 5.6%. Gupta et al. (2015) reported the employment of thermo-alkalistable laccases and xylanases of *Bacillus* sp. and *B. halodurans* in bio-bleaching of pulp as well as in deinking of old newsprints. Of several *Bacillus* spp., *B*. *stearothermophilus* SDX (Dhiman et al., 2008), *Bacillus subtilis* (Annamalai et al., 2009), *Bacillus* strain Sam3 (Chaiyaso et al., 2011), *P. campinasensis* BL11 (Ko et al., 2010), *Bacillus* spp. (Kamble and Jadhav, 2012), and *B. halodurans* (Kumar et al., 2013a) have been identified for alkaliphilic endoxylanases and successfully employed in several industrial applications.

### Acidophilic Endoxylanases

Acidic xylanases find application majorly in baking, juice, and feed industries. Fungi are well known for the secretion of several acidic xylanases. However, due to their short life span under acidic conditions and high molecular weight, bacteria and archaea were also explored for acidophilic xylanases. *B. circulans* D1 (pHopt. 5.0; Bocchini et al., 2008), *Paenibacillus macerans* IIPSP3 (pHopt. 4.5; Dheeran et al., 2012), and *Streptomyces mexicanus* HY-14 (pHopt. 5.5; Kim et al., 2014) represent highly acidophilic xylanases with an optimum activity at or near 5.0 pH. Interestingly, the xylanases produced from *P*. *macerans* IIPSP3 and *S. mexicanus* HY-14 both were recovered from insect guts themselves in an acidic environment, and therefore could reflect the inherent characteristics in their enzymes. The endoxylanase of *S. mexicanus* HY-14 was successfully employed for the generation of XOs from birchwood xylan, while the high molecular weight (205 kDa) endoxylanase of *P. macerans* IIPSP3 may find applicability in bioethanol production as well as in paper bleaching due to two pH optima, i.e., “pH 4.5 and pH 9.0” (Dheeran et al., 2012). Compost soil-derived xylanases also show acidic behavior. Three xylanases have been recovered from compost soil metagenome showing their optimum pH (5.0–6.5) under the acidic region. Here, metagenomic xylanase (MXyl) derived from compost soil shows an exceptional behavior by exhibiting optimum pH of 9.0 (Verma et al., 2013b). Besides, endoxylanases produced from *Thermotoga thermarum* (Shi et al., 2014), *B. subtilis* CHO40 (Khandeparker et al., 2011), *Caulobacter crescentus* (Graciano et al., 2015), and *Bacillus pumilus* B20 (Geetha and Gunasekaran, 2017) also find their optimum pH at around 6.0. The majority of these xylanases have been employed for XO production.

### Halophilic Endoxylanases

Halophilic and halotolerant enzymes have immense applications in various industries. Several downstream processes of industries such as the bioethanol production and paper industries occur in high salt concentration; therefore, more halophilic enzymes are required (Amoozegar et al., 2019). Usually, these enzymes come along with alkaliphilic or thermophilic properties that make them more suitable for industries and therefore exhibit polyextremophilic properties. Halotolerant/halophilic hemicellulases find utility in almost every industry (Setati, 2010). Several bacteria have been reported for the presence of halophilic endoxylanases (Hung et al., 2011; Ghadikolaei et al., 2019). Guo et al. (2009) reported the first salt-tolerant xylanase from a marine bacterium *Glaciecola mesophila* KMM that was able to retain 90% of activity at a higher concentration of 2.5 M NaCl. Similarly, a novel GH-10 family halophilic and thermostable endoxylanase reported from another marine bacterium *Thermoanaerobacterium saccharolyticum* NTOU1 found its optimum activity at 12.5% NaCl (w/v) concentration (Hung et al., 2011). A more halophilic xylanase produced from a halophilic alkali-tolerant bacterium *Chromohalobacter* sp. TPSV 101 showed its optimum activity at 20% NaCl (w/v) under a highly alkaline pH of 9.0 (Prakash et al., 2009). Therefore, this polyextremophilic enzyme may be employed in the seafood processing and juice industries. In a conventional approach of xylanase production from a novel halophilic bacterium, 15% NaCl (w/v) yielded maximum xylanase production, where the endoxylanase was of halophilic properties (Sanghvi et al., 2014). Novel halophilic xylanase was obtained from a halophilic marine bacterium *Marinimicrobium* sp. LS-A18. XylM18. This recombinant xylanase (XylM18) exhibited weak activity in the absence of salt but found stimulation in overall activity in the presence of NaCl up to the concentration of 25% (w/v) (Yu et al., 2019). Similarly, the recombinant xylanase (rXynAHJ14) from *Bacillus* sp. HJ14 retained 62% xylanase activity at a wide range of salt concentrations of 3–30% (Zhou et al., 2014). Such enzymes find suitability in the processing of seafood/feed and best fit for the aquaculture industry. An extracellular endoxylanase from *G. thermodenitrificans* A333 showed fair activity up to 3M NaCl by retaining 70% residual activity for 1 h (Marcolongo et al., 2015). Recombinant xylanase (Xyn11-1) exhibited high salt-tolerant activity by retaining 77.4% residual activity at 0.25–4 M NaCl concentration (Wang et al., 2017). This enzyme is the first report of GH-11 xylanase that was obtained from saline-alkali soil with polyextremophilic properties. Therefore, they may find several industrial applications.

### Psychrophilic Endoxylanases

A very few psychrophilic xylanases have been reported from cold habitats (Van Petegem et al., 2003; Dornez et al., 2011; Wang et al., 2011, 2012; Chen et al., 2013; Qiu et al., 2017). Cold active xylanases have significance in several industries such as food processing, textile, oil extraction, and juice industries (Shallom and Shoham, 2003; Collins et al., 2006). The psychrophilic xylanases have been employed successfully in increasing the dough volume of bread in baking industries (Dornez et al., 2011). The process of dough resting requires cold-active xylanases that can hydrolyze the hemicellulosic content of the bread and improve the quality of the bread by improving the softness, sweetness, and aroma (Dornez et al., 2011; Chen et al., 2013). One of the previous reports describes eight psychrophilic xylanases from the arctic region. Of them, one cold-active xylanase (xyn8) of *Pseudoalteromonas* sp. showed unique features by exhibiting specificity toward xylan only and devoid of activity on aryl-β-glycosides of xylobiose or xylotriose (Collins et al., 2005). All eight enzymes found applicability in the baking industry. Several psychrophilic enzymes including xylanase and β-xylosidase were obtained from a psychrotroph (*Clostridium* strain PXYL1) isolated from a cold-adapted cattle manure biogas digester. This endoxylanase revealed optimum activity at 20°C with a fair stability at 20°C for 2 h (Akila and Chandra, 2003). A novel cold-active xylanase-encoding gene (*xyn27*) was retrieved from metagenomic DNA of frozen soil in Daxinganling, China, which revealed optimum activity at 35°C along with 60.25 and 38.70% residual activity at 20 and 10°C, respectively, under neutral pH having xylobiose as a major product (Qiu et al., 2017). Further characterization of this enzyme showed its multimetal tolerance activity and proposed its application in the food processing industry (Qiu et al., 2017). The members of Bacteriodetes are considered as a good reservoir of hemicellulose-degrading enzymes. *Flavobacterium johsoniae*, a Bacteriodetes, was isolated from mosquito larvae and explored for hemicellulase enzyme activity (Chen et al., 2013). A cold-active xylanase (xyn10A) was isolated from this bacterium that exhibited best activity at 30°C under alkaline pH of 8.0. This modular enzyme was able to retain 50% of its total activity at 4°C. Another novel cold-active xylanase (xynA) from *Sorangium cellulosum* found its maximum activity in the range of 30–35°C under neutral pH which successfully retained ∼33% of its total activity at 5°C (Wang et al., 2012). A halophilic cold-active xylanase (XynA) was characterized from a marine Gram-negative aerobic bacterium (*Zunongwangia profunda*) which also showed optimum activity at 30°C under slightly acidic conditions (Liu et al., 2014). The enzyme activity was increased by 180% in 3 M NaCl, which retained 100% activity at 5 M NaCl. Sequence analysis unveiled novel xylanase by showing a maximum identity of 42.7% only with xylanase of *Bacillus* sp. SN5. Compared to the mesophilic and thermophilic endoxylanases, psychrophilic xylanase count is less and needs to be further explored as the majority of the psychrophilic xylanases showed its optimum activity at or above 30°C.

## Archaeal Endoxylanases

Archaea may be considered as a synonym of extremophiles due to their abundance in extreme habitats (Cabrera and Blamey, 2018). This group of extreme prokaryotes has also been explored for recovering hemicellulases especially thermophilic endoxylanases (Verma and Satyanarayana, 2020). Two strains of *Thermophilum* were the first to be reported from archaeal isolates for having putative hemicellulase activity (Bragger et al., 1989). However, these enzymes were not extensively characterized for their biophysical and biochemical properties. Therefore, xylanases produced from *Thermococcus zilligii* strain A1 may be considered as the first report on characterized endoxylanases (Uhl and Daniel, 1999). The count of archaeal xylanases is comparatively low from bacterial xylanases due to several reasons such as (i) presence of low biomass of archaeal communities in an environmental sample, (ii) inappropriate cultivation strategies for isolating archaea, (iii) inefficient capability to utilize xylan substrate as a carbon source, and (iv) inaccessibility to obtain unculturable archaeal candidates. Therefore, archaeal biology is comparatively tedious to handle as compared to bacterial ones. However, a handful of reports on archaeal xylanases encourage to find more candidates from the extreme environment of archaeal origin. The endoxylanase of *Thermococcus zilligii* strain A1 was highly thermophilic, having a half-life of 8 min at 100°C (Uhl and Daniel, 1999). The endoxylanase was xylan specific and did not show activity toward other polysaccharides. Further analysis on this xylanase revealed that the N-terminal sequence of this xylanase was more similar to the N-terminal sequence of *T. zilligii* maltodextrin phosphorylase (Rolland et al., 2002). Wainø and Ingvorsen (2003) further claimed the first characterization of thermophilic hemicellulases of archaeal origin. This archaeon has been identified as *Halorhabdus utahensis* that showed xylanase as well as β-xylosidase activities. On characterization, both of these enzymes showed their temperature optima in the hyperthermophilic range, i.e., above 60°C. Besides, both shared halophilic properties by having activity up to 30% NaCl (w/v) concentration. Similarly, a hyperthermophilic archaeon *Pyrodictium abyssi* produced an extremely thermostable xylanase that showed its activity at 105°C–110°C. It is one of the most thermophilic xylanases reported so far from any archaeal sources (Andrade et al., 2001). Several polysaccharides have been used as an inducer for the production of xylanase as well as β-xylosidases. Sunna and Antranikian (1997) reported problems for not utilizing xylan substrates by several archaeal isolates, so pretreated xylan, i.e., autoclaved xylan, was used to enrich the medium for isolating xylanase-producing archaea. However, in recent years several archaea have been isolated by using untreated xylan as a sole carbon source (Kublanov et al., 2009; Gavrilov et al., 2016). Such archaea were explored for harnessing endoxylanases. An archaea *Thermococcus* sp. strain 2319x1 was successfully obtained by using sole xylan as a carbon source and showed optimum growth at 85°C under neutral pH. A novel glycosidase gene of *Thermococcus* sp. strain 2319x1 was heterologously cloned and expressed in *E. coli* that revealed five domain structures, where three were coding glycoside hydrolases and two were carbohydrate-binding modules. Therefore, this protein was of very high molecular weight along with broad substrate activity on various polysaccharides (Gavrilov et al., 2016). It has been observed that archaeal xylanases exhibit highly thermostable characteristics that are suitable to withstand the extreme condition of the industries; however, these xylanases are active in either neutral or acidic regions. To the best of our knowledge, no archaeal xylanase has been reported to have alkaliphilic properties to date. When the majority of xylanases used in industries are of either fungal or bacterial origin, the employment of archaeal endoxylanase in industries is still very far with such limitations. With several protein engineering tools, the existing archaeal endoxylanases can be improved for their shelf-life and make them alkaliphilic for their true applications in industries.

## Extremophilic Xylanases of Unculturable Sources

Direct cloning of the community DNA and their analysis for recovering the product of interest made this technology a milestone invention in the field of microbiology. Several xylanolytic enzymes from mesophilic to extremophilic environments have been discovered by using metagenomic (functional and sequence-based) approaches (Verma and Satyanarayana, 2020). The CAZy database shows the presence of approximately 500 candidates of GH-10 and GH-11 xylanolytic enzymes using this approach to date, where the majority of them are uncharacterized. The share of the GH-10 metagenomic representative is almost four-fold as compared to the total count of GH-11 homologs. It indicates the wide distribution of GH-10 xylanases in the environment because these enzymes are less substrate-specific as compared to the GH-11 xylanases. The compost soil metagenome has come out with several hyperthermophilic endoxylanases that showed their optimum temperature beyond 65°C (Verma and Satyanarayana, 2020). For example, metagenomic endoxylanases XYL38 (Ellila et al., 2019), Mxyl (Verma et al., 2013b), and Xyn11 (Kanokratana et al., 2014) recovered from compost metagenome showed the optimum temperature at 80°C. Other sources such as Arctic Mid-Ocean Ridge vent (Fredriksen et al., 2019), Lobios Hot Spring sediment (Knapik et al., 2019), and sugarcane bagasse (Kanokratana et al., 2014) also revealed endoxylanases of hyper-thermophilic nature by having optimum activity at 80°C. The genomic walking PCR (GWPCR) approach uncovered an exceptionally hyper-thermophilic GH-10 endoxylanase from a hot pool metagenome that exhibited an optimum temperature of 100°C under acidic conditions (Sunna and Bergquist, 2003). It was supposed to be a member of Thermotogales endoxylanases; however, its metagenome did not amplify any of their representatives. Of the other extremophilic endoxylanases, metagenomics has also revealed several endoxylanases of acidophilic characteristics that exhibited their optimum pH below 6.0 (Kwon et al., 2010; Wang et al., 2015; Kim et al., 2018; Fredriksen et al., 2019), where the majority of them belong to the GH-10 family. A handful alkaliphilic endoxylanases have also been identified using the nonconventional metagenomic approaches, where most of them exhibit their optimum pH at or near 8.0 (Hu et al., 2008; Thidarat et al., 2012; Ariaeenejad et al., 2019). Only one endoxylanase (Mxyl) showed its optimum activity at pH 9.0 (Verma et al., 2013b). Moreover, this xylanase was of high molecular weight (∼43 kDa); it was even classified into the GH-11 family due to their amino acid composition/hydrophobic cluster analysis and also represent the signature β-jelly-shaped structure of GH-11 endoxylanases. Termite gut acts as a reservoir of plant-degrading bacteria; therefore, the termite gut metagenome has also been explored to harvest endoxylanases (Rashamuse et al., 2016; Liu et al., 2019; Romero Victorica et al., 2020). In a pioneer report, Brennan et al. (2004) reported three GH-8 endoxylanases and one GH-11 endoxylanase from termite gut metagenome that was thermophilic (Topt. 50°C) under an acidic pH range of 5–6. Very similar endoxylanases (Topt. 50°C; pH 5–6) have been reported from other termite gut metagenomes (Rashamuse et al., 2016; Liu et al., 2019; Romero Victorica et al., 2020). While Sheng et al. (2015) reported one cold-active xylanase (xynGH11-7) from the termite gut metagenome that exhibited optimum activity at 30°C under acidic conditions. The majority of the termite gut endoxylanases are acidic and thermophilic in nature which could be due to the acquired characteristics of the gut environments. In a recent investigation, the termite gut metagenome was explored to identify the inhabitant bacterial communities of the termite gut (Romero Victorica et al., 2020). The investigation revealed the presence of five dominant phyla (Firmicutes, Proteobacteria, Spirochaetes, Fibrobacteres, and Bacteroidetes) in the gut of the termites that usually engaged either amino acid (*C. fulviceps*; a grass-wood feeder) or carbohydrate (*N. aquilinus*; a wood feeder) metabolism. The cattle rumen metagenome has also been extensively used for retrieving the extremophilic endoxylanases (Wang et al., 2015; Duque et al., 2018; Kim et al., 2018; Ariaeenejad et al., 2019). Interestingly, the majority of these xylanases showed their optimum temperature at 50°C under acidic (Duque et al., 2018; Kim et al., 2018; Ghadikolaei et al., 2019) as well as alkaliphilic (Ariaeenejad et al., 2019) conditions.

Functional screening of the metagenomic libraries is comparatively more common for identifying the desired clones over the shotgun sequencing-based approaches. However, shotgun-based approaches have also uncovered a few extremophilic endoxylanases (Ghadikolaei et al., 2019; Kumar et al., 2019). Shotgun sequencing of *Kinema* metagenome revealed several industrial enzymes including xylanase deacetylase and β-xylosidase deacetylase. Therefore, Kinema could be employed as a potential source of microbial enzymes used in food processing industries. The 454 pyrosequencing of camel rumen metagenome discovered an acidic and thermophilic GH endoxylanase (XylCMS) with an unusual high molecular weight of 46 kDa (Ghadikolaei et al., 2019). This highly halophilic endoxylanase showed stimulation of 132% in the presence of 5 M NaCl concentration (Ghadikolaei et al., 2019). Analysis of a metagenomic data set from Arctic Mid-Ocean Ridge vent also unveiled a novel thermo-acidophilic endoxylanase (AMOR_GH10A) that showed its optimum activity at 80°C (Fredriksen et al., 2019). This bifunctional endoxylanase (AMOR_GH10A) exhibits binding affinity toward xylan as well as glycan (Fredriksen et al., 2019). Similarly, metagenomic xylanase (UX66) exhibiting exception features by having two carbohydrates and two catalytic domains (Zhao et al., 2010). Mesophilic endoxylanases and like enzymes are countless using the metagenomic approach. Although the reports through shotgun metagenomic approaches on endoxylanases are very less, the technique has huge potential to recover more xylanases. The major concern with these existing metagenomic endoxylanases is their employability in various applications. The majority of these xylanases have not been characterized for their applications under extreme conditions, which leaves a legitimate gap to claim their industrial suitability and needs to be characterized.

## Molecular Attributes to Improve Extremophilic Endoxylanases

Several mechanisms have been laid down to further improve the characteristics of extremophilic xylanases. The majority of the reports discuss the thermostability of the xylanases that include amino acid composition, oligomerization, disulfide bridges, hydrogen bonds, hydrophobic interactions, and inclusion of aromatic amino acids. Several attempts have been made to understand the molecular insights of the extremophilic endoxylanases using protein engineering approaches.

Modular organization and oligomerization have been observed to be one of the major reasons for achieving thermostability among proteins/enzymes (Vieille and Zeikus, 2001). For example, xylanase (XynA) of *T*. *maritima* is organized into five domains, where domains N1 and N2 were found to be crucial for optimum thermostability (Winterhalter et al., 1995). Similar domains have been observed in thermostable xylanase of *Thermoanaerobacterium saccharolyticum* B6A-RI, where deletion of the N-terminal region lost the stability of the xylanase without affecting its catalytic behavior (Lee et al., 1993). The authors suggest that deletion of the N-terminal region may affect the tertiary structure of the xylanase that maintains the stability at the higher temperature.

Amino acid composition finds a significant role in stabilizing xylanases at higher temperatures. Multiple-sequence alignment of mesophilic xylanases with their thermophilic homologs revealed that arginine-rich surfaces contribute to improving the thermostability of the xylanases (Mrabet et al., 1992; Paes et al., 2012). Sriprang et al. (2006) improved the thermostability of thermophilic GH-11 xylanase by 18–20-fold by replacing the surface serine and threonine residues with arginine. Similarly, four cumulative mutations of serine/threonine with arginine residues have significantly enhanced the thermostability of metagenomic xylanase at 90°C (Verma and Satyanarayana, 2013a). The introduction of at least five arginine residues in endoxylanase of *Trichoderma reesei* resulted in a shift in optimum temperature as well as pH (Turunen et al., 2002), while Ayadi et al. (2015) obtained significant improvement in thermostability by replacing serine with threonine residues. Molecular modeling revealed that such mutations (S80T and S149T) assisted in hydrogen bonding and exhibited a packing effect (Ayadi et al., 2015). Hydrogen bonding between S208-N205 and S210-A55 in GH-11 xylanase of *Neocallimastix patriciarum* contributed significantly to improve the thermostability at the higher temperature of 70°C (Han et al., 2017). A double mutant (S22E/N32D) of xylanase protein of *B. subtilis* revealed strong hydrogen bonding for the stabilization of the protein at the higher temperature (Alponti et al., 2016). Therefore, amino acid composition is crucial for enhancing the stability of proteins and enzymes. Few studies indicate that higher alanine content may play a role in the stabilization by making the protein more helical (Vieille and Zeikus, 2001).

Disulfide bridges enhance the stability of proteins by reducing the entropy of the unfolded proteins. Several attempts have been successfully done to improve the thermostability of xylanases by introducing disulfide bonds (Gruber et al., 1998; Turunen et al., 2002; Yang et al., 2017). The half-life of mutated xylanase (Q162H/Q162Y, N11D, and N38E) was increased up to 100 min by introducing disulfide bonds (Turunen et al., 2002). Several such cases have been observed where disulfide bonds along with other favorable mutations improved the thermostability up to 5,000-fold (Xiong et al., 2004; He et al., 2019). Disulfide bonds and multiple-proline substitutions in acidic xylanase of *Aspergillus sulphureus* enhanced the thermostability by 22-fold at 60°C (Yang et al., 2017). The introduction of intra- and intermolecular disulfide bonds in xylanase of *B. circulans* showed a significant improvement in thermostability without any alteration in enzymatic activity (Wakarchuk et al., 1994). Tatu et al. (1993) emphasized that the cleavage of disulfide bonds can reduce 25% β-sheet structure and thus play a significant role in maintaining structural integrity. X-ray crystallographic studies of thermophilic xylanases *T. lanuginosus* and their comparison with several mesophilic homologs revealed the presence of extra disulfide bonds and abundance of charged amino acids throughout the protein (Gruber et al., 1998). β-Elimination of disulfide bridges and cysteine oxidation participate in inhibition and stimulation of the enzyme activity, respectively. β-Mercaptoethanol (β-ME) and dithiothreitol (DTT)-like compounds protect the oxidation of cysteine and therefore either sustain or stimulate the enzyme activity (Knob and Carmona, 2010), while DTT or β-ME has shown a significant reduction in xylanase activity in the presence of DTT or β-ME such as Mxyl (Verma et al., 2013b), XylB8 (Matteotti et al., 2012), and thermostable xylanase of *Talaromyces thermophilus* (Maalej et al., 2009). Such disruptions significantly alter the conformation of the structure required for the functionality of the enzyme (Knob and Carmona, 2010).

Hydrophobic or electrostatic interactions have also been reported to enhance the stability of the xylanases. Thermostable xylanase of *Bacillus strain* D3 was speculated to have thermostability even at a temperature of 75°C due to the hydrophobic interaction, as no cysteine residues were identified that can make them stable like the xylanase of *B. circulans* (73% sequence identity) (Harris et al., 1997). The xylanase of *Bacillus* strain D3 has been studied for thermostabilization, where the presence of cysteine residue may be considered for the formation of disulfide bonds. Moreover, homology modeling revealed the abundance of aromatic amino acids on the surface of the protein that assists in the formation of hydrophobic clusters by which protein exhibited thermostability (Connerton et al., 1999). Hakulinen et al. (2003) also emphasized aromatic residues on the surfaces of the xylanases for improving thermostability. The GH-11 xylanase of *Dictyoglomus thermophilum* also shared a significant portion of polar residues along with a slightly extended C-terminal region for enhanced thermostability (McCarthy et al., 2000).

Similar mechanisms participate in deciding the optimum pH of xylanases to make them acidic or alkaline. Mamo et al. (2009) reported that Val169, Ile170, and Asp171 in the ambiance of acid/base catalytic grooves participate to cleave the xylan residues under alkaline conditions. Moreover, such alkaliphilic xylanases harbored the greater count of acidic residues over the neutral xylanases. In another interesting report, a single amino acid substitution (N35D) in xylanase (BCX) of *B. circulans* was found sufficient to shift the pH optimum toward a more acidic region (5.7–4.6) along with a 20% increase in enzymatic activity. Overall, the study concluded that Asp35 and Glu172 participate in the usual acid/base catalysis mechanism of the enzymes and follow the reverse protonation mechanism (Joshi et al., 2000). Two mutants (E135V and E135R) showed a shift toward alkaline pH by 0.5 and 1.0 units respectively in the GH-11 xylanase of *Bacillus* sp. SN5 (Bai et al., 2016). Structural analysis of these mutants revealed that mutation at the position E135 with valine and arginine residues reduced the overall negative charge on the surface and introduced the salt bridge in the eight-residue loop (Gln131-Pro-Ser-Ile-**Glu135**-Gly-Thr-Ala138) to improve the alkalophilicity of the enzyme. Substitution of arginine at the surface level has been reported in many xylanases to make the xylanase more alkaliphilic (Shirai et al., 1997; Umemoto et al., 2009). Therefore, a golden rule may be proposed that the surface of acidophilic xylanases is rich in acidic residues (Fushinobu et al., 1998; Collins et al., 2005) while alkaliphilic xylanases prefer positively charged amino acids on their surfaces (Shirai et al., 1997; Wu et al., 2020). Such information may be explored to alter the alkalophilicity or acidophilicity of xylanases by determining the overall ratio of acidic or basic amino acids on their surfaces.

Halophilic xylanases also exhibit a greater number of acidic residues on the surfaces of the xylanases (Warden et al., 2015). Wu et al. (2020) reported that halotolerant xylanase (*Ak*XynC) comprised merely 2.73% of positively charged amino acids. Moreover, hydrophobic amino acids also contribute to making xylanase more halophilic (Oren, 2011).

The psychrophilic xylanases exhibit different mechanisms to show maximum activity under low temperature. Van Petegem et al. (2003) described that cold-active xylanases exhibit high flexibility and transformations at low energy costs. The crystal structure of cold-active xylanase (xyn8) from *Pseudoalteromonas haloplanktis* explained that a smaller number of salt bridges and abundance of hydrophobic residue could make an enzyme cold-active (Van Petegem et al., 2003). Zheng et al. (2016) also affirmed that the synergistic effect of a higher number of flexible loops, flexibility in substrate binding residues, and increased exposure of hydrophobic residues enhance the overall activity of cold-active xylanase.

## Applications of Extremophilic Xylanases

Xylanases find applications in different industries such as bakery, paper, food, feed, oil extraction, textile, papad making, juice, biorefinery, prebiotic production, and pharmaceutical industries. By looking into the various steps of downstream processing of these industries, the raw material needs to be gone through several extreme conditions that may include higher temperature, alkaline/acidic conditions, high salt, or solvent-rich solutions. Therefore, the industries look for polyextremophilic endoxylanases that can tolerate such conditions.

### Paper Industry

Thermo-alkali-stable xylanases facilitate the release of lignin from the pulp samples by loosening the multiple cross-linked xylan residues into the lignocellulosic biomass. This process occurs at very high temperatures under alkaline conditions; therefore, xylanases that exhibit stability at higher temperature and pH can perform better over the mesophilic homologs. Polyextremophilic xylanase of *B. halodurans* TSEV1 was successfully employed to bleach the paper pulp where a reduction of 2.42 units in kappa number was observed (Kumar and Satyanarayana, 2012). An alkali-tolerant xylanase of *B*. *licheniformis* 77-2 showed ∼30% reduction in chlorine consumption as compared to the control samples (Damiano et al., 2003). Thermo-alkali-stable metagenomic xylanase (Mxyl) also showed applicability in bio-bleaching of pulp samples where a 29% reduction of chlorine derivatives was observed to achieve an optimum brightness of the paper (Verma and Satyanarayana, 2013b). In an earlier report, the crude broth having thermophilic xylanase from *B. coagulans* was employed to reduce the kappa number by 5.45% of eucalyptus pulp samples (Choudhury et al., 2006). Similarly, a cocktail of thermostable xylanase and pectinase reduced the kappa number by 6.8% and thus reduce the chlorine consumption by 25% without compromising the brightness of the paper (Kaur et al., 2010). The thermophilic xylanases from *B. halodurans* C-125 (Lin et al., 2013) and *B. stearothermophilus* SDX (Garg et al., 2011) were also employed effectively in the bio-bleaching of wheat straw pulp samples. Thermo-alkali-stable xylanases not only reduce the release of chlorine and their derivatives from pulp samples during bio-bleaching but also decrease the dioxin formation that usually generates in a chemical reaction of chlorine and lignin compounds (Sharma et al., 2007). Dioxins are neurotoxic and persistent that exhibit the phenomenon of biomagnification and therefore induce pollution. Thermo-alkali-stable xylanases facilitate the release of lignin from the pulp samples by loosening the multiple cross-linked xylan residues into the lignocellulosic biomass. This process occurs at very high temperatures under alkaline conditions; therefore, xylanases that exhibit stability at higher temperature and pH can perform better over the mesophilic homologs. Polyextremophilic xylanase of *B. halodurans* TSEV1 was successfully employed to bleach the paper pulp where a reduction of 2.42 units in kappa number was observed (Kumar and Satyanarayana, 2012). An alkali-tolerant xylanase of *B*. *licheniformis* 77-2 showed ∼30% reduction in chlorine consumption as compared to the control samples (Damiano et al., 2003). In an earlier report, the crude broth having thermophilic xylanase from *B. coagulans* was employed to reduce the kappa number by 5.45% of eucalyptus pulp samples (Choudhury et al., 2006). Similarly, a cocktail of thermostable xylanase and pectinase reduced the kappa number by 6.8% and thus reduce the chlorine consumption by 25% without compromising the brightness of the paper (Kaur et al., 2010). The thermophilic xylanases from *B. halodurans* C-125 (Lin et al., 2013) and *Bacillus stearothermophilus* SDX (Garg et al., 2011) were also employed effectively in the bio-bleaching of wheat straw pulp samples.

### Generation of Xylo-Oligosaccharides

The use of thermophilic xylanases is also well documented for the generation of XOs from lignocellulosic biomass or agro residues. Hyperthermophilic xylanases of several *Bacillus* spp. (*B*. *halodurnas*, *B*. *amyloliquefaciens*, and *B*. *halodurnas* TSEV1) and *Geobacillus* spp. (*G. thermoleovorans*, *G. thermodenitrificans* TSAAI, and *G. thermoleovorans* AP07) have been successfully employed for the generation of XOs. The majority of these xylanases were endo-acting, therefore producing majorly xylobiose (XO2) along with a significant amount of XO3, XO4, and XO5 oligomers. Xylo-oligosaccharides find fabulous applications in prebiotics by modulating the gut microbiome for their betterment. Cold active halophilic xylanases of *Glaceocola mesophila* KMM 241 (Guo et al., 2009) and *Sorangium cellulosum* (Wang et al., 2012) can be better used for the generation of XOs. Thermophilic and psychrophilic xylanases not only exhibit the saccharification activity but also were able to minimize the possibility of microbial contamination during the process; therefore, they are considered superior over the mesophilic homologs.

### Bakery

Xylanase treatment improves the quality of bread as it reduces the staling rate and increases the shelf-life (Liu et al., 2017; Passarinho et al., 2019). Staling causes significant financial loss to the baking industry. Loss of freshness in terms of lesser moisture increased crumb firmness, and decreased crumb elasticity corresponds to staling. Xylanases are usually incorporated before the baking of dough that assists in the conversion of insoluble hemicellulose into soluble sugars to enhance the sweetness and flavor. Xylanase treatment softens the dough, while softness is the result of arabinoxylan breakdown, which in turn releases water molecules (Oliveira et al., 2014). The development of a synergistic enzyme cocktail is an emerging trend in the baking industry for improved baking products. The selection of enzyme cocktails for the baking industry must be backed by rheological experiments, nutritional and chemical analyses, and baking trials (Almeida and Chang, 2012). In general, such cocktails comprise amylases, xylanases, and lipases exclusively for bread making (Katina et al., 2006). In a study where gluten-degrading enzymes were used along with polysaccharide-hydrolyzing enzymes (amylases and xylanases), bread with better shape was obtained. The polysaccharide-degrading enzymes combine with crosslink-promoting enzymes (glucose oxidase and transglutaminase), an improvement in rheological properties and texture of the bread was recorded (Caballero et al., 2007). Combinations of xylanases and oxidases are used more often as they affect the structure and function of xylan. In some cases, the application of laccase and transglutaminase has also been reported in association with xylanases, where the laccase catalyzes dimerization of feruloylated esters in feruloylated arabinoxylans in the dough (Renzetti et al., 2010). Several studies have shown the successful utility of xylanolytic enzymes in enhancing the quality of the bread, individually or in synergism with other carbohydrases (α-amylase, glucanase, arabinofuranosidase, and laccase) (Flander et al., 2008). *Trametes hirsuta* xylanase and laccase were simultaneously applied on oat–wheat, which resulted in the dough’s softness and an increase in dough volume. This significant improvement of the dough quality is the result of an increase in water content, soluble arabinoxylan, and other water-soluble polysaccharides (Oliveira et al., 2014). Enzyme cocktail of xylanase, β-xylosidase, α-L-arabinofuranosidase, β-glucosidase, avicelase, α-amylase, amyloglucosidase, CMCase, and protease was tested on water-insoluble arabinoxylan for their valuable impact in bread-making. This study showed a beneficial effect of using an enzyme cocktail for the baking industry to improve the quality of bread. Overall, it reduced the amylopectin retro-gradation by 17%, while the crumb firmness was reduced by 25%, and the wheat bread-specific volume increased by 22% (Oliveira et al., 2014). Studies are discussing the role of cold-active xylanases from different microorganisms (Dornez et al., 2011; Wang et al., 2012; Xu et al., 2014). In one such study, bread volume increased up to 28% when cold-active xylanases were used (Dornez et al., 2011). As compared to the mesophilic counterparts, cold-active enzymes display optimal activity at lower temperatures (Huston, 2008). Therefore, psychrophilic enzymes are in high demand in the baking industry, where dough mixing and proofing operate below 35°C. Several investigations have shown the significant effect of low doses of psychrophilic xylanases to attain the maximal bread volume over the mesophilic xylanases (Dornez et al., 2011; Ma et al., 2018).

### Juice Industry

The quality of fruits and vegetable juices can be determined by physically looking for turbidity in juices. For citrus fruits and other juices, turbidity acts as a quality indicator. Haze formation is another area of concern in the juice industry where hazy juice products are undesirable because of customer preferences. The haze consisted of insoluble multimolecular structures formed because of protein–polyphenol interactions (Rai et al., 2003; Pinelo et al., 2010). To remove undesired turbidity and haziness, plant cell wall-degrading enzymes can be employed. These enzymes include pectinolytic, cellulolytic, and hemicellulolytic enzymes. The application of plant cell wall-degrading enzymes results in quality products that appeal to manufacturers and consumers (Adiguzel et al., 2019; Suryawanshi et al., 2019). The use of xylanases for clarification and extraction of fruit juice is gaining momentum (Dhiman et al., 2011; Adiguzel et al., 2019). An alkali-thermophilic cellulase-free xylanase of *Bacillus stearothermophilus* showed potential application in the clarification of citrus juices (Dhiman et al., 2011). When xylanase was used for extraction and clarification of citrus fruit juice, there was a two-fold increase in reducing sugar content, while turbidity was reduced by 35.4% and a 53% increase was observed in the yield of fruit juice after its enzymatic treatment. The apple, sweet lime and pineapple, and pulp were also treated with the thermostable and acid-stable xylanase obtained from *B. licheniformis* to enhance their clarity by Bajaj and Manhas (2012). Treatment with xylanase results in a two-fold increase in the reducing sugar content in apple juice (750–1320 mg/ml), pineapple juice (375–700 mg/ml), and lime juice (300–620 mg/ml). Further, turbidity was reduced by 79% for apple, 70% for pineapple, and 76% for lime juice. Similarly, pineapple, apple, and tomato juices improved multifold when the purified xylanase obtained from *Bacillus pumilus* has been utilized (Nagar et al., 2013). This enzymatic treatment of pulp showed a reduction in turbidity and viscosity without affecting the acid neutrality and significant improvement in juice clarity. In another study, xylanase from *Bacillus pumilus* was used for tomato juice extraction and clarification (Nagar et al., 2012).

### Xylanases in Edible Oil Extraction

Mechanical pressing yields lower oil, while solvent extraction using hexane is not environment friendly which prompted to the development of alternative edible oil extraction processes that permit high oil yield and are eco-friendly. The aqueous enzymatic extraction (AEE) process of oil is an environment-friendly alternative process of vegetable oil extraction. Enzyme-assisted pressing and total solubilization of the oil-bearing constituents have been used in AEE. Cellulases, hemicellulases, and pectinases are the most commonly used cell wall-degrading enzymes used in AEE. Enzyme-assisted pressing employs a mixture of cellulases and hemicellulases. Combinations of cellulases and hemicellulases are determined by the biochemical composition of the secondary cell walls, primary cell wall, and the oil storage cell organelle oleosomes. The cell wall of different oil feedstocks has a different biochemical composition of hemicelluloses and celluloses, organized in a complex structure. In the aqueous enzymatic extraction (AEE) process, water is used as a medium of oil extraction from the feedstock. AEE exploits the amphipathic property of lipid molecules. Water-soluble components of amphipathic lipid molecules interact with a water molecule to form an oil emulsion. This emulsified product from the AEE process is later de-emulsified using physical changes or by adding enzymes to the aqueous medium. It is imminent to know the plant cell wall constituents to be able to select the best enzymes for efficient and optimal oil yields. In the absence of enzymes, the AEE process gives a low oil yield ranging from 28 to 66%. The application of enzymes in AEE enhances oil yield (Dickey et al., 2008). In a recently published study, it has been shown that ultrasonic-microwave-assisted aqueous enzymatic extraction (UMAAEE) can be a quick and environmentally friendly method for oil production from cherry seed oil as compared to the organic solvent extraction method (Hu et al., 2019). Cellulase, hemicellulase, and pectinase in 1:1:1 was used as an enzyme cocktail.

### Other Miscellaneous Applications of Extremophilic Endoxylanases

Xylanases were also employed in wafer improvement. A single report discusses the application of xylanase for refining the quality of black gram-based papad (an Indian traditional wafer) (Awalgaonkar et al., 2015). The water requirement and hardness of dough were significantly reduced by adding 50 mg/kg xylanase into the prepared dough. The black gram contains substantial amount of arabinoxylan; xylanase-based hydrolysis of arabinoxylan also reduced the oil consumption at a significant level.

Due to outstanding hydrolysis of xylanases and cellulases of lignocellulosic biomass (LCB), these enzymes have been successfully employed in biorefinery industries (Smith et al., 2017; Bhardwaj et al., 2019). Disintegration of complex LCB generates monomeric sugars that can be transformed into bioethanol using suitable microorganisms (Basit et al., 2018). Simultaneous scarification and fermentation of LCB in the presence of *Geobacillus* sp. DUSELR13 and *Geobacillus thermoglucosidasius* produced 3.53 and 3.72 g/l ethanol from prairie cord grass and corn stover, respectively (Bibra et al., 2018). Yasuda et al. (2014) also reported a high yield of 74% ethanol by using the SSCF process in the presence of cellulase and xylanase. The majority of the reports suggest the pretreatment of the lignocellulosic biomass using either anhydrous ammonia, acetic acid, or steam to enhance the efficiency of xylanases and cellulases in saccharification (Yasuda et al., 2014; Bondesson and Galbe, 2016). For biorefinery, broad substrate specificity is usually recommended which is a characteristic feature of GH-10 xylanases; however, a report suggests that the xylooligosaccharides may inhibit cellulase and thus reduce the bioethanol yield (Kumar and Wyman, 2009; Qing et al., 2010). The GH-11 xylanases are therefore preferred that are xylan specific and release the sugars in a controlled manner (Henrissat and Davies, 1997; Qing et al., 2010). GH-11 xylanase (XYL10C-ΔN) supports this hypothesis that showed a synergistic effect with cellulose and generates the sugars by reducing the sugar-producing rate to reduce the xylan-induced obstacles (You et al., 2018). Partial utilization of LCB-generated sugars by native microorganisms is one of the limiting factors that could be overcome by developing recombinant strains that can transform the available sugar in an efficient manner (Aftab et al., 2018).

Xylitol is a reduced form of xylose and finds application in pharmaceutical industries especially in oral health. It is the chief constituent of several xylitol-based-chewing gums, toothpaste, and antidiabetic food supplements as a sweetening agent along with anti-cariogenic additive (Chattopadhyay et al., 2014; Rafiqul et al., 2014). In an earlier report, D-xylose sugars were achieved by treating wheat straw with acid and xylanase that yielded 7% xylitol of 99% purity (Liavoga et al., 2007). Several such reports discuss the applicability of xylanase for xylitol production using microbial xylanases (Guo et al., 2013; Kalim et al., 2015; Sena et al., 2017). However, xylose exhibits significant inhibition on xylanase activity which may be up to 80% in the presence of 10% (w/v) xylose. Therefore, xylitol production demands stringent conditions that also include thermostable xylanases during the chemo-catalytic hydrogenation process. A novel one-pot cascade process yielded 80% xylitol in the presence of xylanase and ruthenium/carbon catalyst (Ayubi et al., 2021). The estimated market of xylitol is $740 million which encourages the wide applications of thermophilic and barophilic endoxylanase to achieve highly valued chemical xylitol and improving the lignocellulose variolization.

## Conclusion

The estimated global industrial enzyme market was approximately US$ 5.6 billion during 2019, and it is projected to rise at a compound annual growth rate (CAGR) of 6.4% from 2020 to 2027,^2^ where xylanolytic enzymes share a significant chunk of total carbohydrases. Therefore, more efforts should be made for presenting endoxylanases and like enzymes as an alternative to chemical and physical methods to make the environment eco-friendlier cost-effectively. The GH families (GH-5, GH-7, GH-8, GH-10, GH-11, GH-43, and GH-141) harbor several extremophilic xylanolytic enzymes of potential biotechnological applications. Extremophilic xylanases not only enhance the bioconversion of the lignocellulosic biomass into a valuable product but also reduce the possibilities of microbial contamination during the entire process. Bacterial endoxylanases may be considered as the better option for industrial applications over the eukaryotic sources because of their broad range of properties at extreme pH and temperatures. The existing archaeal endoxylanases exhibit hyperthermophilic properties; however, the majority of them are either acidic or neutral. Therefore, protein engineering approaches can be used for improving their alkalophilicity. Even directed evolution, site-directed mutagenesis, and DNA shuffling approaches may be considered for extending the half-life of several bacterial xylanases that exhibit their optimum temperature at or above 80°C but lose their stability at these temperatures for longer incubations. Metagenomic approaches have not been well explored to date for harnessing extremophilic endoxylanases that exhibit the potential to reveal novel candidates from extreme environments. Therefore, functional as well as shotgun-sequenced-based approaches can be employed for retrieving novel targets. Moreover, the microbiome analysis of the environmental samples should be assessed to go with a rational approach that may enhance the discovery of a novel target multifold by correlating their metabolic profiling with the inhabitant microorganisms of that environment. A significant effort should also be made to characterize the existing xylanolytic enzymes where their count is significantly high. Commercialization of endoxylanases needs to be boosted by emphasizing their unique characteristics to the industries as well as for the sake of the environment.

## Author Contributions

DV conceptualized and wrote the article.

## Conflict of Interest

The author declares that the research was conducted in the absence of any commercial or financial relationships that could be construed as a potential conflict of interest.

## Publisher’s Note

All claims expressed in this article are solely those of the authors and do not necessarily represent those of their affiliated organizations, or those of the publisher, the editors and the reviewers. Any product that may be evaluated in this article, or claim that may be made by its manufacturer, is not guaranteed or endorsed by the publisher.

## Footnotes

1
www.cazy.org

2
www.grandviewresearch.com
